# Antibacterial and Antifungal Properties of Electrospun Recycled PET Polymeric Fibers Functionalized with Zinc Oxide Nanoparticles

**DOI:** 10.3390/polym13213763

**Published:** 2021-10-30

**Authors:** Katherine Vázquez, Paul Vanegas, Christian Cruzat, Néstor Novoa, Ramón Arrué, Eulalia Vanegas

**Affiliations:** 1 Chemical Engineering, Faculty of Chemical Sciences, University of Cuenca, Cuenca 010203, Ecuador; katherine.vazquez@ucuenca.edu.ec; 2 Department of Space and Population, Faculty of Chemical Sciences, University of Cuenca, Cuenca 010203, Ecuador; paul.vanegas@ucuenca.edu.ec; 3 Center for Environmental Studies, Department of Applied Chemisty and Production Systems, Faculty of Chemical Sciences, University of Cuenca, Cuenca 010203, Ecuador; christian.cruzat@ucuenca.edu.ec; 4 Laboratorio de Química Inorgánica y Organometálica, Departamento de Química Analítica e Inorgánica, Facultad de Ciencias Químicas, Universidad de Concepción, Edmundo Larenas 129, Casilla 160-C, Concepción 4070386, Chile; nenovoa@udec.cl (N.N.); rarrue@udec.cl (R.A.)

**Keywords:** recycled poly (ethylene terephthalate), electrospinning, zinc oxide nanoparticles, antibacterial activity, antifungal activity

## Abstract

Currently, to reduce the environmental problems associated with plastic waste, methods are being sought to use this waste as raw materials in different applications, such as fibers. In addition, to improve these materials and provide different properties, nanoparticles (NPs) are incorporated. In the present work, polymeric fibers made of recycled polyethylene terephthalate (r-PET) from post-consumer water bottles, functionalized with 0%, 1.5%, 3% and 6% zinc oxide nanoparticles (ZnO-NPs) in function of r-PET weight, were elaborated to evaluate their antibacterial and antifungal characteristics. The ZnO-NPs were synthesized by the solvothermal method, obtaining particles with a mean diameter of 38.15 nm, while the fibers were obtained by electrospinning with a diameter range between 200–5000 nm. The functionalized fibers were carried out against *Escherichia coli* and *Bacillus subtilis* through the agar diffusion method, obtaining the highest inhibition halo at 6% w/w ZnO-NPs, being 26.5 mm and 34.25 mm, respectively. In addition, the same method was used to evaluate the antifungal activity of *Penicillium s.p.* and *Fusarium graminearum*, observing antifungal properties due to the presence of nanoparticles in the fibers.

## 1. Introduction

Currently, there is global concern about the contamination produced by the high amount of plastic waste, primarily those of petrochemical origin [1] such as polycarbonate (PC), polypropylene (PP), polyethylene terephthalate (PET), polyvinyl chloride (PVC), polystyrene (PS), styrene copolymers (acrylonitrile butadiene styrene (ABS), styrene acrylonitrile (SAN), acrylonitrile styrene acrylate (ASA)), and so forth [2,3]. The massive contamination caused by plastic waste is due to its high resistance to atmospheric and biological agents, and its decomposition in the environment lasts for years [4].

Global estimates denote an abrupt growth in plastic consumption from 1.5 million tons (Mt) in 1950 to 330 Mt in 2015. Approximately 5% of global plastic consumption corresponds to Latin America (16.5 Mt), from which 18% (2.97 Mt) represents the amount of PET in all its application areas, with 35% (1.04 Mt) used in the packaging industry. Surprisingly in Latin America, it is estimated that only 14% (0.14 Mt) of plastic waste is recycled [5], whereas the remaining percentage ends up in clandestine landfills, controlled landfills, water bodies, vacant lots or incinerators [6,7]. In addition, the large amount of this waste stream is linked to the high demand for PET plastic bottles, since this material presents characteristics that makes it suitable for storing and carrying different types of liquid, from water and soft drinks to oil [8]. The issue is not the weight of the waste but the volume occupied by the discarded bottles, since one ton comprises roughly 20,000 units of 250 mL bottles (including caps) [9].

Due to their low density and high resistance to biodegradation, PET bottles are easily moved by water or gusts of wind and end up contaminating the soil and oceans, threatening the habitats and health of terrestrial and aquatic species. The conventional approach followed by several countries is the recycling of this waste stream to reduce its environmental impact. Nonetheless, recycling rates are still low, even in countries with advanced waste management systems and significant experience in recycling [10].

The ideal option would be reprocessing the plastic waste to manufacture new bottles, but its use is restricted to the first use in case of contamination by chemicals or by the high level deterioration of its mechanical properties, in particular, the thermodegradable degeneration of acetaldehyde in PET material [11]. For these reasons, several groups are seeking to develop new products based on PET using recycling as a practical option, where discarded bottles serve as raw materials for manufacturing of new value-added products [12], thereby encouraging their pickup on a larger scale [13].

Recycled PET (r-PET), similar to PET, stands out for its resistance to corrosion and chemicals, and its low density and availability [14]. Thus, r-PET could be an option as a replacement for virgin PET in a variety of specialized applications that use different manufacturing processes [15]. Nanofibers stand out for their large surface area, surface flexibility, mechanical performance and controllable pore structures [16,17]. For the purpose of performance in different application areas, nanofibers have been introduced into sensors [18], tissue engineering [19], textiles [20], filtration media [13], drug release [21], the automobile industry [22,23], household items, and sporting goods [24,25], among others.

There are different methods to produce fibers, where electrospinning is the most popular technique for the production of nanofibers from polymer solutions [18,26]. As an advantage, continuous ultrafine fibers [20] with diameters ranging from micrometers to several nanometers are obtained using this method, and are suitable for the incorporation of nanoparticles (NPs) or nanocomposites [27]. Furthermore, electrospinning is a simple yet versatile method, since it allows the development of fibers with different morphologies, including thread, hole, core-shell, and others [28]. In addition, it allows the possibility of recycling used solvents through condensing units coupled to the process [13].

The addition of NPs to the fibers provides new properties such as antimicrobial activity [29]. This biocidal action is achieved through the incorporation of different compounds to a support matrix (fibers) such as transition metal ions, which can damage cell membranes or alter enzymatic functions, leading to the inhibition of DNA replication, and eventually bacteria, viruses or fungi die [30,31,32].

The most used inorganic compounds that stand out as antibacterial agents are: noble metals, transition metals, and their derivatives (oxides), mainly those based on silver, copper, zinc, cobalt and titanium [33]. A key factor to consider during the selection of an antibacterial agent is cost, which is why metals such as Cu, Co, Zn or Ti are selected over Ag despite the latter being a highly effective and well-studied agent [34]. Zinc oxide nanoparticles (ZnO-NPs) are highlighted as biocides because they are biocompatible and non-toxic for humans [35] and can also be synthesized in different morphologies, including nanowires, nanobuds, nanoparticles, and nanorods.

Considering this context and the opportunity presented by the potential use of r-PET to reduce environmental impacts by obtaining new value-added products, this work includes the use of r-PET in the synthesis of ZnO-NPs/r-PET composite fibers using the electrospinning method. The addition of ZnO-NPs allowed an antimicrobial effect, which was evaluated by antibiograms. These functionalized composites could offer solutions to common microbial problems in textiles for medical or surgical purposes such as aprons, among others.

## 2. Materials and Methods

### 2.1. Materials

PET from the “Cielo” brand post-consumer water bottles (r-PET) with a capacity of 625 mL was used for the fiber preparation; Trifluoroacetic acid (TFA, for analysis) was purchased from Merck. For the synthesis of ZnO-NPs, zinc (Zn, powder) from Sigma-Aldrich (St. Louis, MO, USA), nitric acid (HNO_3_, purity: 65%) and sodium hydroxide (NaOH), both Merck brand, and absolute ethanol from J.T.Baker, USA (C_2_H_5_OH, purity: 99.9%) were used. For the antimicrobial tests, the bacteria Bacillus subtilis (Gram positive) and Escherichia coli (Gram negative), and the fungi Fusarium graminearum and Penicillium s.p. were provided by the Laboratory of Agricultural Sciences of the University of Cuenca, Ecuador.

### 2.2. Methods

#### 2.2.1. Synthesis of ZnO-NPs

Zinc oxide nanoparticles (ZnO-NPs) were synthesized by the solvothermal method; 1.23 g of zinc metallic powder was dissolved in 15 mL of HNO_3_ and added to 20 mL of ethanol, and 10 mL of 0.8 M NaOH was added dropwise with constant stirring. The resulting 30 mL of the above mix solution was deposited in a 45 mL Teflon reactor (Parr Instruments 4744, Moline, IL, USA), sealed and placed inside a Shel Lab SVAC1 oven (USA) and heated at 150 °C for 21 h at constant temperature. The ZnO-NPs were then cooled to room temperature and washed four times in a sonicator (Branson Ultrasonics M 1800-E, Danbury, CT, USA): three times with distilled water, separating them in a centrifuge (20 min, 1000 rpm) and filtered off. Then, the last wash was performed with ethanol (50 min, 1000 rpm). Subsequently, the solid material was dried under vacuum in an Shel Lab SVAC1 oven at 60 °C for 8 h.

#### 2.2.2. Characterization of Nanoparticles

The morphology of ZnO-NPs was characterized by Transmission Electron Microscopy (TEM) using a JEOL-JEM 1200EXII, Peabody, Massachusetts, USA equipment with 4 Å resolution. The previous preparation of the sample was performed by placing the solid material on a dry copper grid (150 mesh) previously coated with carbon in inert atmosphere.

#### 2.2.3. Electrospinning Stage

The fibers were fabricated on the electrospinning equipment Tong Li Tech TL-01 (Shenzhen, China) at room temperature, with constant movement in the *X*-axis and a 22-gauge metal needle having an internal diameter of 0.7 mm and a distance to the drum of 12 cm. The applied voltage was 20 kV and a feed flow of 3.5 mL/h. The electrospun fibers were collected on the rotating roller coated with aluminum foil.

#### 2.2.4. Polymeric Solutions for Electrospinning

The r-PET solution was prepared with a concentration of 20% (*w*/*v*) in TFA. The bottle was cut into pieces for subsequent weighing on a Sartorius Entris 224I-1S (Goettingen, Germany) analytical balance and then the TFA solvent was added. The solution was magnetically stirred on a Corning PC-420D magnetic stirrer (Mexico) at 300 rpm until a homogeneous solution was obtained. The ZnO-NPs were added once the r-PET was completely dissolved in TFA by constant magnetic stirring (150 rpm) for 1–2 h (the time lapse depends on ZnO-NPs’ concentration). Four fibers were obtained with 0%, 1.5%, 3% and 6% w/w of ZnO-NPs and were coded as r-PET, ZnO-1.5/r-PET, ZnO-3/r-PET, and ZnO-6/r-PET, respectively.

#### 2.2.5. Characterization of Electrospun Fibers

The Energy Dispersive X-ray Spectroscopy (EDS) analysis was performed using a Bruker X-flash 630 detector (Baden-Württemberg, Germany) with a resolution of 123 eV inside the Scanning Electron Microscopy (SEM) chamber in an approximate area of 104 μm × 104 μm. The fibers were characterized by SEM using TESCAN MIRA 3 equipment (Tescan, Brno, South Moravian, Czech Republic), for which the sample was coated with approximately 20 nm of gold (99.99% purity) with a Quorum Q150R sputtering evaporator (Quorum Technologies, Lewes, UK) and using a secondary electron detector with an accelerating voltage of 10 kV. In addition, Differential Scanning Calorimetry (DSC) experiments were carried out on a STAR Default Db Mettler Toledo (Mettler Toledo, Milan, Italy), to characterize temperature transition in each material subject to the same condition. Temperature ranged from 20 °C to 290 °C, with a heating rate of 10 °C/min.

#### 2.2.6. Antibacterial and Antifungal Activity Tests

A Gram-positive bacterium (*Bacillus subtilis*) and a Gram-negative bacterium (*Escherichia coli*) were used for the antibacterial analyses, whereas the fungi *Fusarium graminearum* and *Penicillium s.p.* were used in the antifungal experiments. For both cases, the modified Kirby–Bauer disk diffusion method [36] was used for a qualitative evaluation of antimicrobial capacity in each composite. Before undergoing analysis, samples were irradiated with UV light to avoid interferences and false positives. The size of the four samples was 1 cm × 1 cm, which were placed on top of the agar plates inoculated with swabs by sweeping the surface with the bacteria first in one direction and then in the opposite direction to cover the entire agar surface. They were then incubated (considering parameters by gender of the microorganism), to finally measure the zone of inhibition present on the back of the plate with a ruler, for the case of bacteria, while for antifungal properties, fungal growth path was taken into account to evaluate the effect due to the presence of r-PET composite fiber [37,38,39].

## 3. Results and Discussion

### 3.1. Synthesis and Characterization of ZnO-NPs

**Figure 1:**
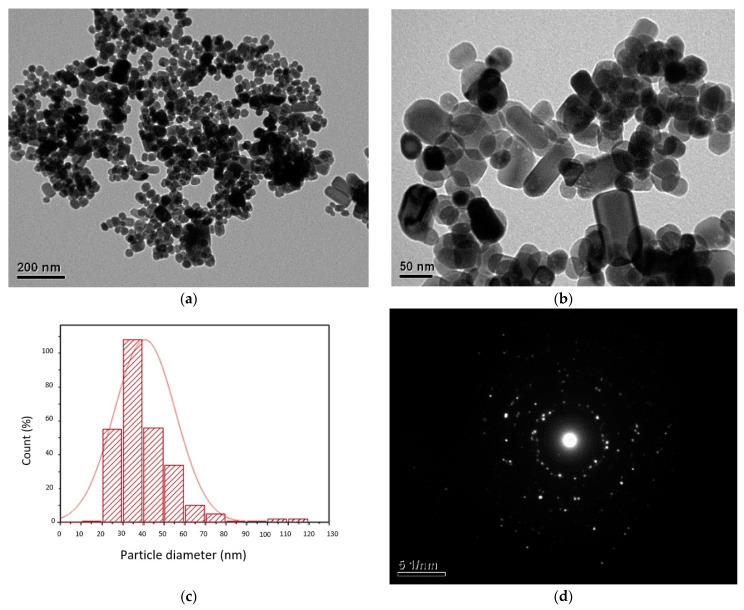
(**a**,**b**) TEM micrograph of ZnO-NPs; (**c**) particle size distribution of ZnO-NPs; and (**d**) EDS patterns of ZnO-NPs.

Once the reaction time had elapsed, the ZnO-NPs were dried and weighed, obtaining an average weight of 0.6 g by each experiment. The prepared nanoparticles had white color, and formed small agglomerates but not powder-like dispersed particles. This phenomenon is mainly due to the high surface area of the NPs and the strong attraction between the ZnO-NPs [40]. The TEM analysis revealed that the ZnO-NPs show various shapes, including spheres, hexagons, and ovals, although some of them showed an elongation in their structure, as can be seen in Figure 1a,b. Moreover, Figure 1c exhibits the morphology of the ZnO-NPs with an average size of 38 nm, which confirms the nanosize of the obtained material; also, the particle size dispersion had a value ranging between 19 nm and 119 nm.

Figure 1d shows the pattern of very sharp dots formed by electron diffraction allowing the identification of crystallographic features, and corroborates the crystallinity of the authentified ZnO-NPs. Additionally, the EDS analysis of these ZnO-NPs allows establishing a crystallographic system belonging to the hexagonal crystalline system, possibly corresponding to a wurzite-type structure [41].

### 3.2. Characterization of r-PET Composite Fibers

**Figure 2:**
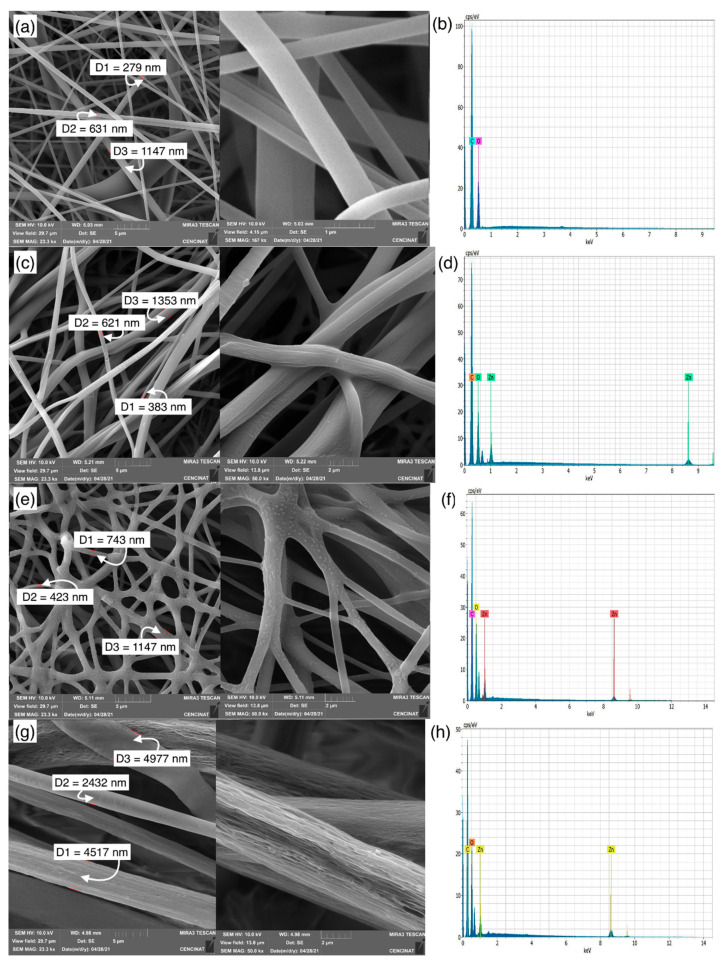
SEM analysis and EDS spectrum of samples: (**a**,**b**) r-PET; (**c**,**d**) ZnO-1.5/r-PET; (**e**,**f**) ZnO-3/r-PET; (**g**,**h**) ZnO-6/r-PET, respectively.

The proposed methodology allowed the obtention of flexible, easy to handle and resistant polymeric fibers (sheets). These sheets presented an approximate size of 16 cm x 16 cm, which varied at the moment of removing them from the aluminum foil, since some samples presented a strong adherence which prevented a correct detachment and generated fiber loss. The weight of the samples varied also for this reason, ranging between 2.7–2.8 g. SEM analysis was performed to examine the morphology of the composites obtained from r-PET fibers functionalized with ZnO nanoparticles (Figure 2); a significant difference between runs was observed, such as the surface smoothness, beads presence, and diameter uniformity of the resulting fibers. Figure 2a shows the micrograph of the r-PET polymeric fiber, where a smooth morphology can be observed, i.e., smooth interlaced fibers that form the polymeric sheet, whose diameters range between 279 nm and 1147 nm. These results are consistent with those reported by other research groups [18,42,43]. On the other hand, Figure 2c,e,g depict the SEM analysis of samples ZnO-1.5/r-PET, ZnO-3/r-PET, and ZnO-6/r-PET, respectively. The fiber diameter ranges between 383–1353 nm (ZnO-1.5/r-PET), 423–1690 nm (ZnO-3/r-PET 1), and 2432–4977 nm (ZnO-6/r-PET), establishing that as the solution concentration increases by the addition of the nanoparticles, the fiber diameter increases; that is, the higher the concentration, the viscosity increases [42]. Furthermore, their wide variation indicates that the fiber diameters are not uniform. However, this resulted in smooth-surfaced, randomly oriented, soft and flexible ZnO-NPs/r-PET functionalized fibers. Although the concentration of the solution influences the morphology of the fiber [43,44,45], the viscosity of the solution can be attributed to being the main cause of these changes in the morphology of the electrospun fiber [46]. At high polymer concentration and viscosity, the jet is less prone to breakage due to higher viscoelastic interactions, resulting in continuous fibers. However, when the viscosity limit is exceeded, the flow through the needle is interrupted, causing clogging [47,48].

Fiber structure and diameter can be precisely controlled by experimental details, including viscosity, electric field strength and distance from the needle tip to the collector. Solutions with lower polymeric concentration have lower viscosity; thus, as the jet travels towards the collector, the low viscosity promotes jet extension and results in thinner fibers [49]. At small distances and a constant voltage, the electrostatic field is stronger; therefore, it exerts greater electrostatic forces on the jet and elongates the fibers. Simultaneously, the strong field causes a higher mass flow which tends to increase the fiber diameter. These effects reverse at higher distances, and the fibers have more time to elongate [13].

Establishing a relationship between solution concentration and diameter, Zhang et al. (2011) reported that at a PET concentration of 12.8 wt%, an average nanofiber diameter of approximately 600–1000 nm was obtained. As the solution concentration decreased, so did the diameter; when it was reduced to 9.5% by weight, the nanofiber diameter was approximately 300–500 nm, whereas with a 6.5% by weight, the average diameter of the fiber was reduced to about 200–400 nm. The decrease of viscosity is due to the lower concentration of the polymer [50]. Therefore, the wide variation in the diameter of the fibers could be attributed to a non-uniform jet expulsion, caused by the high viscosity derived from the increase in the concentration of ZnO-NPs. It could also attributed to strong movements required during the cleaning processes, which could reposition the needle at a different distance from the initial parameters [51].

Figure 2c shows the SEM analysis for ZnO-1.5/r-PET sample; this micrograph shows a mat-like shape whose yarns presented folds (roughness) on the surface, but no beads.

For the ZnO-3/r-PET an interlaced fibrous mat can be observed in Figure 2e. The presence of beads on the surface is notorious, which could represent the ZnO-NPs agglomeration on the surface. When the concentration of ZnO-NPs changed to 6% *w*/*w*, roughness is observed on the fiber surface (Figure 2g). The significant variation in the diameter and morphology of the fiber is eventually due to the concentration of ZnO-NPs. Furthermore, an additional experiment was attempted with a concentration of 9% w/w, but this was not achieved due to the high viscosity of the solution. This parameter prevented the passage through the hose that connects the syringe with the metal tip. It should also be taken into account that increasing the polymer concentration leads to the formation of larger fiber diameters [52].

On the other hand, the EDS analysis is shown in Figure 2 and its data are recorded in Table 1. The EDS spectrum of r-PET nanofiber (Figure 2b) indicated that the main elements of films consist of carbon and oxygen. In addition, ZnO-1.5/r-PET, ZnO-3/r-PET and ZnO-6/r-PET modified fibers exhibit the EDS spectrum recorded in Figure 2d,f,h, respectively. The peaks around 1 and 8.5 keV show the presence of Zn, confirming that the ZnO-NPs were successfully incorporated into the original r-PET polymer.

**Figure 3:**
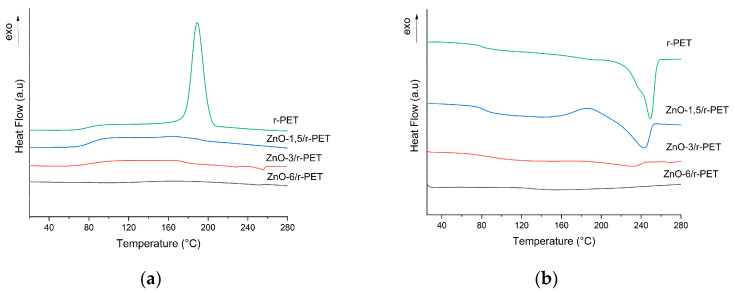
(**a**) Cooling stage thermogram and (**b**) second heating stage thermograms of r-PET composite fibers.

The DSC curves in the cooling scan from melt and subsequent heating are shown in Figure 3. The r-PET nanofiber shows the characteristic temperature transitions of standard PET, during the cooling scan the glass transition temperature (T_g_) was observed at 84 °C and the registered crystallization temperature (T_c_) was around 190 °C, while during the second scan the melting temperature (T_m_) was 249 °C.

The ZnO-NPs’ content and thermal properties are related. For the ZnO-1.5/r-PET fiber, an exothermic peak was observed in the second heating (cold crystallization) [53,54] where the melting temperature of r-PET slightly decreased. The ZnO-NPs hindered the crystallization, resulting in less perfect crystal structures and a lower melting point; r-PET fibers with ZnO-NPs concentration lower than 1.5% were semicrystalline polymers, while PET with higher ZnO-NPs concentration became amorphous, with neither crystallization nor melting behavior in both the cooling and heating scans (Table 2). On the other hand, the T_g_ raised its value with the increment of ZnO-NPs concentration, as the ZnO-NPs restrict the motion of the PET chain [49,55]. 

### 3.3. Antibacterial and Fungal Analysis

For determining the antimicrobial properties of functionalized fibers, NPs-ZnO/r-PET, the analyses were carried out using *Bacillus subtilis* and *Escherichia coli* and the *Fusarium graminearum* and *Penicillium s.p*.

**Figure 4:**
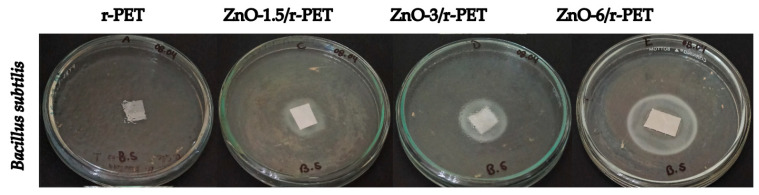
Antibacterial tests Bacillus subtilis in polymer composite fibers.

**Figure 5:**
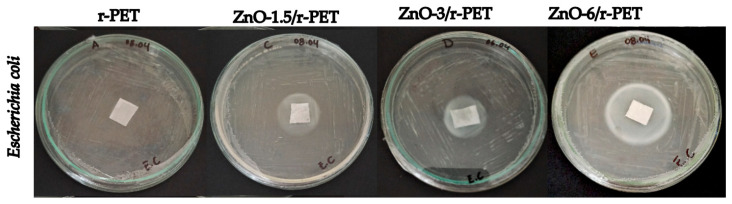
Antibacterial tests Escherichia coli in r-PET composite fibers.

Table 3 and Table 4 show the number of replicates with their respective inhibition halo measurements, obtaining an average of the diameters in millimeters at different percentage of ZnO-NPs load (0; 1.5%; 3%; 6%) against *Bacillus subtilis* (Figure 4) and *Escherichia coli* (Figure 5). It is observed that the antibacterial activity is clearly dependent on the ZnO-NPs concentration for both strains; the zone of inhibition increased at higher concentrations of ZnO-NPs in the polymeric matrix. The inhibition zone was 23.75 mm and 34.25 mm at 1.5 and 6% w/w of ZnO-NPs load for *Bacillus subtilis*, respectively. The exact mechanism of ZnO-NPs inhibition is still a matter of debate among the research community, several authors have proposed: generation of reactive oxygen species (ROS), release of Zn^2+^ ions and accumulation of nanoparticles in the cell membrane or cytoplasmic region [35]. In the release of Zn^2+^ ions mechanism, the ions penetrate the cell wall of bacteria and eventually kill them; this liberation of active Zn^2+^ rises with the concentration of ZnO-NPs in the resulting modified fiber, and thus enhances the inhibitory effect [56,57]. Furthermore, the greatest inhibitory effect was observed against *Escherichia coli* with a zone of inhibition of 38.5 mm diameter followed by *Bacillus subtilis* with a zone of inhibition of 34.25 mm diameter at 6% w/w of ZnO-NPs concentration. Although ZnO-NPs usually have a stronger antimicrobial effect against Gram-positive bacteria such as *Staphylococcus aureus* [56] and *Listeria monocytogenes* [58,59], this study shows that these nanoparticles are also effective against Gram-negative bacteria.

Previous studies have highlighted the use of ZnO nanostructures in different thermoplastic polymers to investigate antimicrobial and antifungal susceptibility [60]. The antimicrobial susceptibility of ZnO-NPs/polyester composite films against *Escherichia coli* and *Staphylococcus aureus* bacteria was observed, where control polyester films did not show any zone of inhibition. However, the ZnO-NPs/polyester composite films had antimicrobial activity and the zone of inhibition was clearly visible. The diameter of the inhibition zone was measured and found to increase with increasing ZnO-NPs concentration. The diameter for *Escherichia coli* was 10, 15.7, 18.2 and 21.2 mm for films loaded with 0.25, 0.50, 0.75 and 1.0 wt% ZnO-NPs, respectively. A similar trend was observed for *Staphylococcus aureus* with a diameter of 15.2, 18.7, 22.6 and 29.0 mm for the films loaded with 0.25, 0.50, 0.75 and 1.0 wt% ZnO-NPs, respectively. As indicated in this investigation, ZnO-NPs produce reactive oxygen species that cause bacterial wall rupture and thus antimicrobial behavior [61].

In other research, the antimicrobial activity of PET and carboxymethylcellulose (CMC) bilayer film containing different concentrations of ZnO-NPs was tested against the bacterial strains *Staphylococcus aureus* and *Escherichia coli*. The results revealed that the antimicrobial properties of ZnO-NPs/PET-CMC were more effective on Gram-positive bacteria than on Gram-negative bacteria. Furthermore, the antimicrobial effects of nanocomposite films of various biopolymers, such as agar and CMC containing ZnO-NPs, were reported. It was found that the addition of ZnO-NPs to the film inhibited the growth of *Listeria monocytogenes* and *Escherichia coli* [62]. The antibacterial properties of ABS and ZnO-NPs/ABS electrospun nanocomposite membranes were compared. ZnO-NPs imparted antimicrobial activity to the ABS electrospun membrane and showed an inhibition zone of 10 and 11 mm in diameter against *Escherichia coli* and *Staphylococcus aureus,* respectively. It is believed that reactive oxygen species such as hydroxyl radicals (•OH), hydrogen peroxides (H_2_O_2_) and singlet oxygen (^1^O_2_) generated by ZnO-NPs lead to the destruction of bacterial cells [63].

Antimicrobial tests were performed using the viable colony count method on poly (lactic acid) (PLA) and ZnO-NPs composite films. As expected, the pure PLA film showed no antimicrobial activity against both bacteria. The antibacterial activity of the composite films was determined against the foodborne pathogenic bacteria *Escherichia coli* and *Listeria monocytogenes*. However, ZnO-NPs/PLA composite films showed distinct antimicrobial activity against *Escherichia coli* and *Listeria monocytogenes*, depending on the type of bacteria and the concentration of ZnO-NPs. As in the control groups (without any film or pure PLA film), the amount of *Listeria monocytogenes* increased until 6 h of incubation in the ZnO-NPs/PLA composite film groups, and thereafter, their growth started to slowly decrease. In the case of *Escherichia coli*, bacterial growth was constant until 3 h, and began to decrease thereafter. The intensity of antibacterial activity depended on the concentration of ZnO-NPs in the composite films. The initial increase in microbial counts with the ZnO-NPs/PLA composite films was presumably due to the slow release of the ZnO-NPs from the PLA matrix [64]. The antibacterial effect of ZnO-NPs/latex coatings against *Colibacillus* and *Staphylococcus aureus* showed that 99% of both were killed with 3 wt% ZnO-NPs [65].

In another study, antimicrobial properties of low-density polyethylene (LDPE) and ZnO-NPs/LDPE nanocomposites were reported. The nanocomposites were around 99.99% effective against *Escherichia coli* after irradiation with UV light. It was reported that H_2_O_2_ generated to UV light can penetrate cells, causing their peroxidation and then their death [66]. The results of antibacterial experiments on high-density polyethylene (HDPE) as ZnO-NPs/HDPE films showed an increase in antibacterial activity with increasing ZnO-NPs content. When the content was 0.5% by weight, the antibacterial rate reached 89.8% against *Escherichia coli* and 95.3% against *Staphylococcus aureus*. The antibacterial rate showed a slow increase when the concentration was higher than 0.5 wt% [67]. On the other hand, electrospun fibers of polyacrylonitrile (PAN, 10% *w*/*w*) and ZnO-NPs (5% *w*/*w*) showed antibacterial activity of 75.65% and 70.05% against *Staphylococcus aureus* and *Pseudomonas aeruginosa* bacteria, respectively [68].

The synthesized ZnO-NPs/r-PET fibers showed profound antibacterial capacity against *Escherichia coli* and *Bacillus subtilis* at different ZnO concentrations, which makes these fibers a great alternative to combat Gram-positive and Gram-negative bacteria. The results confirm that the ZnO-NPs were released from the r-PET matrix and diffused with significant amount to the culture medium, forming bacterial inhibitory halos within the expected range according to the previously cited reference. The results obtained open a promising field to replace other bactericide compounds that could cause bacterial resistance. Therefore, the prepared nanocomposite fibers can be used for food packaging or medical applications. However, it should be noted that some studies have indicated a high toxicity of ZnO-NPs to human cells at relatively high concentrations and low particle sizes [69]. Indeed, ZnO has shown properties against intestinal diseases by protecting intestinal cells from *Escherichia coli* infection by inhibiting bacterial adhesion and internalization [70].

Research has mainly focused on bactericidal mechanisms, so the proposed fungicidal mechanisms of nanoparticles are extrapolated from proposed mechanisms for bacteria leading to enzyme inactivation and death by oxidative stress [71,72]. In the case of fungal organisms, it is possible that ZnO-NPs perturb the membrane lipid bilayer [69]. As for the antifungal effects, both toxigenic fungi (*Fusarium graminearum* and *Penicillium s.p*) were sensitive to the effect of the nanoparticles; similar to the bacterial strains, the fungal growth was significantly reduced at high concentrations of ZnO, using the agar dilution method. Yet, it should be noted that no concentration used completely inhibited the fungal activity, but it seems to depend on the concentration and time of exposure to Zn-NPs [73,74,75,76].

**Figure 6:**
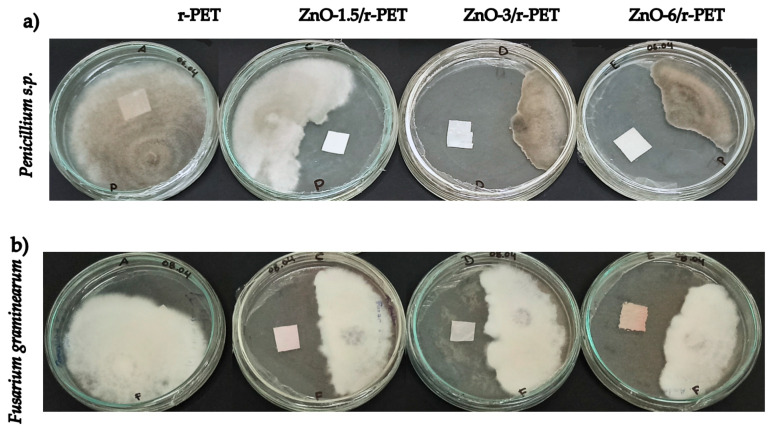
The growth of (**a**) Penicillium s.p and (**b**) Fusarium graminearum in r-PET composite fibers.

In contrast to the antibacterial analysis, the inhibition halo was not measured during the antifungal test; however, it is possible to observe antifungal properties of the functionalized fibers being evident in Figure 6a,b for *Penicillium s.p.* and *Fusarium graminearum*, respectively. A strong fungicidal characteristic is granted, as the concentration of nanoparticles was increased. The highest antifungal effect was observed for ZnO-6/r-PET compared to r-PET, for both fungi. For these results, the research should be continued to elucidate the action of mechanisms of nanoparticles supported into the polymer, as well as to evaluate their potential activity on other pathogenic microorganisms.

## 4. Conclusions

The solvothermal method proved to be a feasible method to obtain ZnO nanoparticles with an average diameter of 38 nm. The fibers obtained via the electrospinning method had diameters ranging between 200 nm and 5000 nm, showing a directly proportional relationship between diameter and nanoparticles concentration, due to the increase in the viscosity of the solution. The ZnO-NPs based r-PET composite fibers have a broad antibacterial and antifungal spectrum. The analyses showed a greater effect against *Escherichia coli* (38.5 mm) than against *Bacillus subtilis* (34.25 mm), while for the fungi chosen, they were more effective against *Penicillium s.p.* than against *Fusarium graminearum*. As the results to date are quite promising, additional research is required around the toxicity mechanisms of the ZnO-NPs based r-PET composite fibers on these and other microorganisms.

## Figures and Tables

**Table 1:** EDS data analysis of r-PET composite fibers.

Element	r-PET		ZnO-1.5/r-PET		ZnO-3/r-PET		ZnO-6/r-PET	
	wt%	at.%	wt%	at.%	wt%	at.%	wt%	at.%
Carbon	63.93	70.24	56.37	64.09	62.48	70.12	54.82	64.15
Oxygen	36.07	29.76	41.57	35.48	34.8	29.32	41.78	35.13
Zinc	-	-	2.06	0.43	2.72	0.56	3.4	0.72

**Table 2:** Thermal properties of r-PET composite fibers.

Sample	T_g_ (°C)	T_c_ (°C)	T_m_ (°C)	X_c_ (%)
r-PET	84	190	249	0.25
ZnO-1.5/r-PET	84	186 *	243	0.10
ZnO-3/r-PET	90	-	-	-
ZnO-6/r-PET	**	-	-	-

* Cold crystallization; ** the thermograms showed no clear glass transition.

**Table 3:** Average diameters in millimeters of the inhibition haloes of Bacillus subtilis bacteria at different concentrations of ZnO-NPs.

Sample	r-PET	ZnO-1.5/r-PET	ZnO-3/r-PET	ZnO-6/r-PET
1	0	23	24	31
2	0	27	23	33
3	0	25	27	39
4	0	20	23	34
Zone of inhibition (mm)	0	23.75 ± 2.58	24.25 ± 1.63	34.25 ± 2.58

**Table 4:** Average diameters in millimeters of the inhibition haloes of Escherichia coli bacteria at different concentrations of ZnO-NPs.

Sample	r-PET	ZnO-1.5/r-PET	ZnO-3/r-PET	ZnO-6/r-PET
1	0	28	28	40
2	0	28	30	36
3	0	27	25	40
4	0	21	25	38
Zone of inhibition (mm)	0	26 ± 2.91	27 ± 2.12	38.5 ± 1.65

## Data Availability

Not applicable.
